# Essential Oils as Plant-Derived Bioherbicides: Prospects for Sustainable Weed Management—A Narrative Review

**DOI:** 10.3390/molecules31193532

**Published:** 2026-10-03

**Authors:** Amra Bratovčić, Juliana Navarro Rocha, Ferdinando Branca, Donata Arena, Anja Vieweger, Milica Aćimović

**Affiliations:** 1 Department of Physical Chemistry and Electrochemistry, Faculty of Technology, University of Tuzla, Urfeta Vejzagića 8, 75000 Tuzla, Bosnia and Herzegovina; amra.bratovcic@untz.ba; 2 Department of Plant Science, Agrifood Research and Technology Centre of Aragon (CITA), Avda. Montanana 930, 50059 Zaragoza, Spain; jnavarroro@cita-aragon.es; 3 Department of Agriculture, Food and Environment, University of Catania, Via Santa Sofia 100, 95123 Catania, Italy; fbranca@unict.it (F.B.); donata.arena@unict.it (D.A.); 4 Department of Crop Sciences, Research Institute of Organic Agriculture FiBL, Ackerstrasse 113, CH-5070 Frick, Switzerland; anja.vieweger@fibl.org; 5 Institute of Field and Vegetable Crops Novi Sad (IFVCNS)—National Institute of the Republic of Serbia, Maksima Gorkog 30, 21000 Novi Sad, Serbia

**Keywords:** phytotoxicity, allelopathy, herbicidal activity

## Abstract

Growing herbicide resistance and concerns regarding the environmental and health impacts of synthetic herbicides have intensified interest in plant-derived weed-management products. This review assesses the phytotoxic, herbicidal, and allelopathic potential of essential oils, emphasizing efficacy, selectivity, formulation, and delivery. Relevant literature was identified through searches of Scopus, Web of Science, and Google Scholar, using terms related to essential oils, phytotoxicity, allelopathy, herbicidal activity, and weed management, followed by critical comparison of selected studies. Essential oils from diverse aromatic plants inhibited weed germination and growth through mechanisms including membrane disruption, pigment degradation, oxidative imbalance, and altered antioxidant activity. Species- and genus-specific evidence highlights the considerable chemical diversity of essential oils and identifies numerous compounds and formulation strategies with potential for bioherbicide development. Activity varied according to chemical composition, concentration, formulation, application conditions, and target species. Nanoemulsions, nanocapsules, and cyclodextrin-based systems have been investigated to improve stability, controlled release, and delivery, although improved formulation properties do not necessarily translate into greater herbicidal efficacy. Although some formulations achieved substantial weed suppression with limited crop injury, reported cytogenotoxicity, effects on soil microbial activity, and phytotoxicity at high concentrations highlight the need for further evaluation of potential non-target effects. The available evidence is still dominated by laboratory studies, while field-scale validation remains limited. Essential-oil-based bioherbicides are promising components of integrated weed management, but their practical development requires standardized formulations, mechanistic studies, field-scale validation, economic assessment, regulatory support, and comprehensive evaluation of crop and environmental safety.

## 1. Introduction

The rapid expansion of herbicide-resistant weed populations, together with growing concerns about the environmental and health impacts of synthetic agrochemicals, has increased the demand for sustainable weed management alternatives. Bioherbicides have therefore been investigated as potential components of integrated weed control systems, using natural compounds or biological agents to suppress weed growth [1,2]. These bio-based options encompass microbial formulations, botanical extracts, essential oils, and isolated phytotoxic metabolites capable of disrupting critical physiological processes, such as cell division, nutrient uptake, photosynthesis, hormonal balance, and oxidative balance [1]. Recent reviews have addressed the mechanisms of bioherbicidal action, formulation strategies, application methods, and prospects for commercial use [1,3,4]. However, the effectiveness of plant-derived bioherbicides can vary considerably under field conditions. Their chemical composition is affected by plant genotype, environmental conditions, extraction procedures, and formulation. Other factors that may limit their practical use include a narrow weed-control spectrum, formulation instability, production costs, potential crop phytotoxicity, and regulatory requirements [1,2,4].

Allelopathy is one of the natural mechanisms underlying plant-mediated weed suppression. It involves chemical interactions between organisms mediated by secondary metabolites, known as allelochemicals, which can affect the germination, growth, survival, and reproduction of neighboring species [3]. Many plant species produce allelochemicals with phytotoxic effects, and their use in agricultural systems through crop rotation, intercropping, cover crops, mulching, green manuring, and direct application of plant extracts has been investigated as a means of reducing reliance on synthetic herbicides [5,6]. Advances in analytical chemistry and biotechnology have facilitated the identification, isolation, and characterization of bioactive phytotoxic metabolites. Phenolic compounds, terpenoids, and alkaloids are among the major classes of allelochemicals associated with phytotoxic activity [7]. Understanding the relationships between chemical composition, modes of action, and biological responses is important for improving the efficacy and performance of plant-derived bioherbicides under field conditions.

Aromatic plants are important sources of phytotoxic secondary metabolites. Their essential oils, extracts, and distillation co-products, including hydrolates and solid residues, contain bioactive compounds, particularly terpenoids and phenolic derivatives, that can inhibit weed seed germination and early seedling growth [8]. Essential oils are of particular interest because their complex mixtures of volatile compounds can affect several physiological processes in plants. Their phytotoxic activity depends on chemical composition, concentration, application conditions, and the susceptibility of the target weed species. Achieving a balance between phytotoxic efficacy and crop safety remains a major challenge in developing essential oils as commercial bioherbicides. Unlike synthetic herbicides designed to act on specific metabolic targets, plant-derived products often show broad-spectrum phytotoxicity and limited systemic movement within plant tissues, which can make selective application in crops difficult. Understanding the mechanisms underlying the different responses of weeds and crops, as well as identifying the compounds or mixtures responsible for these effects, is important for improving selectivity [8]. The increasing prevalence of herbicide-resistant weeds and the limited availability of new synthetic modes of action further increase the need for botanical alternatives [9].

The use of essential oils and other plant-derived bioherbicides under field conditions is limited by variable efficacy, physical instability, high production costs, and complex chemical composition [10]. Recent research has therefore focused on formulation and application strategies that can improve stability, bioactivity, and selectivity. These approaches include advanced delivery systems, combinations of bioactive compounds, and the integration of botanical products into integrated weed management programs. The difference between laboratory and field performance is an important consideration in the development of essential oils and their constituents as bioherbicides. Although key compounds such as carvacrol, thymol, and eugenol show strong phytotoxicity in laboratory tests [11], their herbicidal efficacy under field conditions can vary with target weed phenology, application rate, foliar coverage, and formulation [12]. Overcoming these field-level limitations requires targeted formulation and application strategies that enhance volatile stability and deliver consistent weed control.

## 2. Literature Search and Selection

A narrative literature search was conducted using Scopus, Web of Science, and Google Scholar. The literature search was conducted during July and August 2026 and covered publications from 2010 to August 2026. The search used combinations of the following terms: “essential oil”, “essential oils”, “bioherbicide”, “phytotoxicity”, “allelopathy”, “weed control”, “weed management”, and “herbicidal activity”. The search terms were combined in different ways to identify studies addressing the herbicidal or phytotoxic effects of essential oils, their major constituents, and essential oil-based formulations. Relevant studies were considered, with particular attention to experimental evidence from laboratory, pot, and field studies. Studies focused exclusively on plant extracts or other non-essential-oil materials, as well as studies unrelated to weed management or phytotoxic activity, were not considered. Backward and forward citation tracking of key relevant publications was additionally performed to identify studies not retrieved through the database searches.

## 3. Evidence of Phytotoxic Activity of Essential Oils

Essential oils serve as valuable sources of botanical herbicidal agents owing to their phytochemical diversity and demonstrated phytotoxicity. Their principal constituents have demonstrated phytotoxic activity both as individual compounds and within complex mixtures, providing molecular leads for sustainable weed control products. Over recent decades, integrated experimental approaches have increasingly clarified the physiological and biochemical mechanisms behind essential oil herbicidal activity [13]. The retrieved literature was critically evaluated, and key studies are summarized in Table 1, including information on target weed or cultivated species, experimental conditions (*in vitro*, pot, and field experiments), essential oil composition, and tested concentrations.

The following represents a comprehensive overview of species and genera that have shown allelopathic/phytotoxic activity. The details describe how these properties were determined and how the effects could be utilized and developed in further studies.

### 3.1. Achillea millefolium *L.* (Yarrow)

*Achillea millefolium* essential oil has demonstrated phytotoxic and allelopathic activity against several plant species. *In vitro* application of the essential oil inhibited seed germination and early seedling growth of *Sinapis arvensis, Lolium multiflorum, Raphanus sativus, and Hordeum vulgare*, with *S. arvensis* showing the highest sensitivity [14]. The observed phytotoxicity was also associated with inhibition of *α*-amylase activity during seed germination, suggesting that interference with reserve mobilization may contribute to the inhibitory effects of the essential oil [14]. Similarly, *A. millefolium* essential oil showed allelopathic activity across different plant organs and phenological stages. Despite variation in oil yield and chemical composition, germacrene D, sabinene, borneol, camphor, *α*-pinene, and artemisia ketone were consistently identified among the major constituents, supporting their potential contribution to the observed phytotoxic effects [15]. Evidence for the herbicidal potential of *Achillea* extends beyond essential oils. Soil incorporation of aerial dry biomass from nine *Achillea* species significantly inhibited the growth of *Amaranthus retroflexus*, with *A. vermicularis* and *A. aleppica* causing approximately 90% reduction in weed dry biomass. GC-MS profiling identified 152 essential oil constituents across the investigated species, while partial least squares regression associated *cis*-menth-2-en-1-ol, *α*-terpinyl propionate, and bornyl acetate most strongly with the suppression of *A. retroflexus* [61]. Although this study did not directly evaluate isolated essential oils, the results provide complementary evidence that *Achillea* species contain volatile constituents potentially contributing to their broader phytotoxic and allelopathic activity.

### 3.2. Acorus calamus *L.* (Sweet Flag)

The essential oil of *Acorus calamus* exerts pronounced, family-dependent phytotoxic effects on Fabaceae and Brassicaceae species. Primary root growth inhibition assays revealed IC_50_ values of 0.03% for *Vicia faba*, 0.025% for *Lupinus luteus*, 0.01% for *Brassica napus*, and 0.005% for *Arabidopsis thaliana*. Although exposure at these respective IC_50_ concentrations produced equivalent stress levels, physiological responses diverged significantly between families: Fabaceae accumulated higher H_2_O_2_ levels but mounted a more effective antioxidant response, whereas Brassicaceae experienced severe metabolic disruption, lipid peroxidation, membrane impairment, electrolyte leakage, and reduced cell viability [16]. At the cellular level, treatment at species-specific IC_50_ thresholds triggered oxidative and replication stress, marked by DNA double-strand breaks and delayed S-phase progression. Replication activity declined in all four species, with a higher proportion of cells replicating heterochromatin, indicating a prolonged S-phase. However, these genotoxic insults occurred without inducing endoreplication, apoptotic DNA fragmentation, or widespread cell death [62]. The essential oil also disrupted mitosis across all tested species, decreasing the mitotic index by 50–60% while inducing chromosomal abnormalities, aberrant spindle organization, and structural alterations in microtubule and actin filament networks. Changes in H_3_T_3_ and H_3_S_10_ phosphorylation point to interference with epigenetic regulation during mitosis. Ultimately, these cellular impacts were consistent across species, indicating that selectivity stems from differential dose sensitivity rather than distinct modes of action [63].

### 3.3. Anethum graveolens *L.* (Dill)

The essential oil of *Anethum graveolens* has shown phytotoxic activity against weed species, although the reported effects vary among experimental systems. In a comparative study of essential oils from six aromatic plants, *A. graveolens* oil, characterized by limonene and carvone as its major constituents, significantly inhibited the germination of *Solidago canadensis* seeds [17]. In another study, the essential oils of seven annual aromatic plants were evaluated against *Echinochloa crus-galli*. The oil of *A. graveolens* showed relatively low phytotoxicity compared with those of sweet fennel and sweet basil, which were the most active oils in the assay [18]. More recent work demonstrated substantial variation in the phytotoxicity of *A. graveolens* oils according to the plant part used for distillation. Oils from inflorescences, stems, and leaves, dominated by *α*-phellandrene and *p*-cymene, inhibited the germination and growth of *Sinapis arvensis*, *Trifolium campestre*, and *Lolium multiflorum*, with complete inhibition of germination and seedling growth of *S. arvensis* and *T. campestre* at the highest tested concentrations; IC_50_ values ranged from 0.3 to 1.6 mg/mL [19]. In a recent study of essential oil nanoemulsions, all tested *A. graveolens* nanoemulsion concentrations inhibited weed germination, while post-emergence assays indicated selective herbicidal effects for some of the tested essential oils. The study also identified the D1 protein of photosystem II as a potential target of phytotoxic terpenoids through molecular docking, although the reported selective effects were associated with other essential oils rather than *A. graveolens* [20].

### 3.4. Artemisia *spp.*

The phytotoxic activity of *Artemisia* essential oils has been reported for a range of weed species, with studies also showing that the activity of an oil cannot necessarily be predicted from the abundance of its major constituents. In *A. absinthium* essential oil, linalool was identified by bioassay-guided fractionation as the major contributor to phytotoxic activity, although 1,8-cineole was more abundant in the oil. Linalool inhibited root-hair formation and metaxylem development, indicating that its activity was associated with specific changes in root development [21]. Comparative studies further demonstrated phytotoxic activity of *A. absinthium* and *A. vulgaris* essential oils against *Amaranthus retroflexus* and *Setaria viridis*, with the two oils differing in their relative activity toward the tested weeds [22]. The herbicidal activity of *A. absinthium* was also reported against *Sinapis arvensis* [23]. For *A. vulgaris*, strong inhibition of root growth was subsequently observed in both monocotyledonous and dicotyledonous species, including *S. viridis*, *Poa annua*, *Medicago sativa*, and *A. retroflexus* [30].

Several studies have addressed the processes affected by *Artemisia* oils or their major constituents. *A. austriaca* essential oil and its major monoterpenoids, camphor and 1,8-cineole, inhibited *Avena fatua* germination by affecting hormonal balance and the mobilization of seed reserves [24]. The essential oil of *A. campestris* showed concentration-dependent effects on germination, with inhibition at higher concentrations but increased germination at the lowest concentration tested; *Daucus carota* was the most strongly affected species [25]. In *A. fragrans*, essential oil treatment of *Convolvulus arvensis* impaired photosynthetic performance and was accompanied by oxidative stress and electrolyte leakage in both laboratory and pot experiments [26]. Similarly, *A. scoparia* essential oil inhibited emergence and seedling growth of several weeds and caused visible injury following post-emergence application, together with changes in chlorophyll content, respiration, and membrane integrity [29]. The activity of *Artemisia* essential oils has also been evaluated in relation to crop species and environmental effects. *A. herba-alba* essential oil inhibited germination and radicle elongation of both weeds and crops, while no adverse effects were observed in the three tested aquatic and soil organisms under the experimental conditions [27]. *A. lancea* essential oil inhibited germination and root and shoot growth of *A. retroflexus* and *S. viridis*, with complete inhibition of germination at concentrations of 1 mg/mL and above [28].

### 3.5. Chrysanthemum balsamita *(L.) Baill.* (Costmary)

*Chrysanthemum balsamita* essential oil exhibited pronounced phytotoxic effects on wheat and barley. At 30 and 90 µL, it completely inhibited germination and early seedling development of both species, while at 10 µL, it reduced germination and seedling growth more strongly in barley than in wheat. The essential oil was dominated by camphor (43.7%), *trans*-thujone (32.4%), and camphene (11.6%) [31].

### 3.6. Cinnamomum *spp.*

*Cinnamomum* essential oils have shown pronounced phytotoxic activity in both laboratory and greenhouse experiments. Cinnamon oil completely inhibited seed germination at 1.8 and 5.4 mg L^−1^ under controlled conditions, while 345.6 mg L^−1^ completely suppressed germination of *Amaranthus retroflexus* under greenhouse conditions. Among the three tested essential oils, cinnamon produced the strongest inhibitory effects, although the response was also strongly influenced by the applied concentration [35]. In post-emergence greenhouse assays, cinnamon bark essential oil caused severe injury to a range of weed species within five days, with little or no subsequent recovery and no harvestable biomass 20 days after treatment [34]. Further studies with *Cinnamomum cassia* oil showed rapid increases in malondialdehyde content, electrolyte leakage, and water loss, together with cellular plasmolysis, membrane disruption, and chloroplast damage in treated leaves of *Trifolium incarnatum* and *Lolium perenne* [32]. The activity of *C. cassia* essential oil was also retained after nanoformulation. The nanoemulsion strongly inhibited lettuce radicle growth and caused visible injury and more than 90% desiccation of *Agropyron repens* and *Bromus japonicus* within seven days. In *A. repens*, treatment reduced chlorophyll index, SOD activity, and protein content, while increasing ion leakage. Cinnamaldehyde was the predominant constituent of the oil (86.93%) [33].

Allelopathic effects have also been reported for *Cinnamomum camphora*. Camphor released from leaf material into the soil was identified as the principal allelochemical affecting rice seedling growth, with an additional contribution from camphor absorbed through the stomata from the atmosphere [64]. More recently, *C. camphora* leaves inhibited the vegetative propagation of the invasive weed *Alternanthera philoxeroides*, with stronger effects at higher amounts of leaf material and under temperatures ≥ 30 °C. Camphor and linalool strongly suppressed stem and root vegetative propagation and were associated with increased H_2_O_2_ and MDA accumulation, altered antioxidant enzyme activities, and reduced levels of soluble sugars, proteins, and proline [65].

### 3.7. Coriandrum sativum *L.* (Coriander)

The *Coriandrum sativum* essential oil, rich in 1,8-cineole (36.9%), linalool (28.7%), and limonene (20.2%), displayed moderate phytotoxic activity, inhibiting weed germination and subsequent seedling growth [18].

### 3.8. Cuminum cyminum *L.* (Cumin)

*Cuminum cyminum* essential oil showed the strongest allelopathic activity among the six EOs tested. It completely inhibited germination and seedling development of both wheat and barley at all tested application rates (10, 30, and 90 µL). Its chemical profile was dominated by cuminaldehyde (49.6%), followed by *α*-terpinen-7-al (11.6%), *p*-cymene (10.4%), and *β*-pinene (9.1%) [31].

### 3.9. Cymbopogon *spp.*

Essential oils from *Cymbopogon* species display strong herbicidal activity against both monocotyledonous and dicotyledonous weeds. *Cymbopogon nardus* essential oil markedly suppressed germination and subsequent growth of *Digitaria horizontalis* and *Cenchrus echinatus*, reducing plant height, shoot and root biomass, and chlorophyll and total protein contents. At a 20% concentration, the oil reduced shoot and root biomass and decreased chlorophyll and protein levels by more than 80% and 90%, respectively [38]. Pure citronellal showed stronger germination inhibition than the whole oil, indicating that the phytotoxicity of *C. nardus* essential oil cannot be attributed solely to this constituent. When formulated as a nanoemulsion rich in citronellol (35.2%), geraniol (21.9%), and citronellal (13.6%), *C. nardus* oil caused visible phytotoxic effects in *Echinochloa crus-galli* and *Amaranthus tricolor*, accompanied by increased electrolyte leakage and MDA content and reduced chlorophyll and carotenoid levels [37]. Similarly, *Cymbopogon citratus* essential oil, rich in neral (29.2%) and geranial (18.2%), ranked among the most active oils against *Sinapis arvensis* in comparative screening [23]. In outdoor pot trials, *C. citratus* oil applied alone reduced *Avena sterilis* biomass to 31–33% of the control, whereas its combination with pelargonic acid reduced *Lolium rigidum* dry biomass by 77% [36]. The activity of lemongrass oil was predominantly contact-based, with limited translocation within treated plants, which may restrict the persistence of its herbicidal effect and allow regrowth.

### 3.10. Echinophora cinerea *Boiss.* (Fedaleh)

The essential oil of *Echinophora cinerea* inhibited *Chenopodium album* seed germination and seedling growth in a dose-dependent fashion. Essential oils distilled from various plant parts consistently exhibited phytotoxicity, with chemical profiles dominated by monoterpenes and phenolic monoterpenoids such as *α*-pinene, *γ*-terpinene, linalool, carvacrol, and thymol [39]. Physiologically, exposure to the essential oil induced oxidative stress, leading to elevated H_2_O_2_ accumulation, lipid peroxidation, and electrolyte leakage.

### 3.11. Eucalyptus *spp.*

Leaf essential oils from five *Eucalyptus* species (*E. loxophleba*, *E. bicolor*, *E. amplifolia*, *E. campaspe*, and *E. salubris*) showed distinct compositional profiles, with 1,8-cineole being an important compound differentiating the examined oils [41]. In phytotoxicity bioassays against weeds (*Lolium multiflorum* and *Sinapis arvensis*) and crops (*Raphanus sativus* and *Cucumis sativus*), *E. loxophleba* and *E. salubris* oils showed the strongest inhibition of seed germination and root elongation. Similarly, *Eucalyptus citriodora* essential oil strongly inhibited germination and post-emergence growth of *Digitaria horizontalis* and *Cenchrus echinatus*, reducing plant height, shoot and root biomass, and chlorophyll and protein contents [38]. In another study, *E. citriodora* oil strongly inhibited the invasive weed *Nicotiana glauca* while producing comparatively limited effects on the tested crop species [40]. *Eucalyptus cladocalyx* essential oil, dominated by spathulenol (21.6%), 1,8-cineole (20.5%), and *p*-cymene (15.1%), also ranked among the most active oils against *Sinapis arvensis* [23].

### 3.12. Foeniculum vulgare *Mill.* (Fennel)

*Foeniculum vulgare* essential oil, characterized by estragole/anisole (40.1%) and anethole (38.1%), inhibited *Solidago canadensis* seed germination, with its activity associated particularly with the highly phytotoxic phenylpropanoids [17]. In a previous Petri-dish bioassay involving four weed species (*Portulaca oleracea*, *Chenopodium album*, *Solanum nigrum*, and *Echinochloa crus-galli*), *F. vulgare* essential oil, rich in *trans*-anethole (58.4%) and limonene (16.0%), exhibited pronounced phytotoxicity [18].

### 3.13. Hesperozygis ringens *(Benth.) Epling* (Espanta-Pulga)

The essential oil of *Hesperozygis ringens* suppresses growth across several invasive and weed species. In *Eragrostis plana*, the essential oil inhibited seed germination and seedling growth starting at 0.001%, while foliar exposure in adult plants reduced chlorophyll levels and induced tissue damage linked to hydrogen peroxide accumulation and loss of plasma membrane integrity [45]. Assays with pulegone, the primary constituent, produced weaker germination and seedling inhibition than the whole oil, indicating that its phytotoxicity relies on interactions among multiple constituents. Similarly, *H. ringens* essential oil exerted phytotoxic effects on *Lolium multiflorum* from 0.001%, achieving complete suppression of germination and seedling establishment at 0.01 to 0.02%, alongside reductions in chlorophyll content, root and shoot biomass, and membrane stability [44]. Foliar treatment with 1% essential oil on adult and field-grown *L. multiflorum* caused chlorophyll loss and cellular lesions comparable to those induced by ammonium glufosinate.

In evaluations against *Conyza bonariensis* and the native species *Plantago australis*, application of 0.01 to 0.02% essential oil inhibited germination and early growth, with *C. bonariensis* suffering more severe membrane damage [46]. Pulegone alone exhibited lower toxicity than the complete essential oil. Foliar application on adult plants produced visible injury within 24 h, reaching peak damage at 72 h. Against *Bidens pilosa*, 0.01 to 0.02% essential oil strongly suppressed germination and early development, whereas pulegone alone was less effective [43]. Foliar exposure to 1% essential oil caused visible and anatomical leaf damage in *B. pilosa* within 24 h, while causing only minor effects in the non-target species *Paspalum notatum*. Under greenhouse and field conditions, *H. ringens* essential oil displayed a dose-dependent response against the broadleaf weed *Amaranthus viridis* [42]. Concentrations of 3% and 6% reduced germination and seedling establishment, lowered chlorophyll content and photosynthetic capacity, and elevated electrolyte leakage and tissue damage. In field trials, 6% essential oil induced severe necrosis and complete wilting faster than the commercial herbicide. The oil exhibited higher potency against germinating seeds (EC_50_ of 2.11 to 3.09%) than against established plants (EC_50_ of 2.57 to 5.52%).

### 3.14. Hyssopus officinalis *L.* (Hyssop)

*Hyssopus officinalis* essential oil also exhibited strong allelopathic activity. At 30 and 90 µL, it completely suppressed germination and early seedling development of both wheat and barley. At 10 µL, the essential oil reduced several growth parameters, including radicle number, radicle length, seedling height, and vigor index; however, wheat germination itself was not significantly affected at this concentration [31].

### 3.15. Lavandula *spp.*

Essential oils of *Lavandula* species exert concentration-dependent phytotoxic effects across both weed and crop species. While lavender essential oil suppressed germination in several Mediterranean weeds, its activity was lower than that of cinnamon oil [35]. Among the individual components of *L. angustifolia* oil, terpinen-4-ol, lavandulol, and linalyl acetate strongly inhibited germination and root elongation in *Setaria verticillata*, while combinations of selected constituents with carvacrol or eugenol exhibited synergistic activity [66]. Whole *L. angustifolia* oils have also shown pronounced phytotoxic effects in relation to their chemical composition. An essential oil dominated by linalyl acetate (47.0%) and linalool (28.4%) completely suppressed germination and early seedling growth in wheat and barley at higher application rates [31]. This oil also showed strong activity against *Lolium multiflorum*, whereas tomato and cucumber seedlings exhibited differential sensitivity [40]. Similarly, an oil rich in linalool (34.84%) and menthyl acetate (28.10%) inhibited the growth of both the monocotyledonous weed *Echinochloa crus-galli* and the dicotyledonous *Galium aparine* [47]. However, *L. angustifolia* oil showed contrasting effects in baby-leaf lettuce, with low concentrations stimulating growth, biomass accumulation, photosynthetic pigments, and mineral uptake, whereas a 2.0% application caused marked inhibition [48].

### 3.16. Leptospermum scoparium *J.R. & G. Forst.* (Manuka)

*Leptospermum scoparium* essential oil represents a particularly interesting case among plant-derived bioherbicides because its activity has been associated with leptospermone, a natural *β*-triketone and precursor of synthetic HPPD-inhibiting herbicides. In outdoor pot experiments, manuka oil reduced the biomass of *Lolium rigidum*, *Avena sterilis* and *Galium aparine* by more than 90%, while combinations with pelargonic acid achieved even greater suppression. Although these results suggest a high practical potential compared with many other essential oils, validation under field conditions and crop-safety assessments remain necessary [36].

### 3.17. Levisticum officinale *Koch.* (Lovage)

The essential oil of *Levisticum officinale*, dominated by *α*-terpinyl acetate (64.8%) and *β*-phellandrene (14.7%), primarily affected early seedling development rather than seed germination [31]. Application doses of 10, 30, and 90 µL reduced radicle number, radicle length, seedling height, and vigor index. Across all tested rates, wheat seed germination remained uninhibited, indicating a weaker effect on germination than on post-germination development.

### 3.18. Lippia *spp.*

Essential oils of *Lippia* species display distinct phytotoxic and ecotoxicological profiles driven by their chemotype composition. The eugenol-rich essential oil of *L. alba* selectively suppressed growth in the weeds *Lolium multiflorum* and *Sinapis arvensis* while exerting lower toxicity toward the crops *Hordeum vulgare* and *Raphanus sativus* [49]. This physiological inhibition correlated with reduced *α*-amylase activity and altered catalase activity in treated seedlings. In evaluations of *L. origanoides* essential oil, where camphor was the primary constituent among high levels of oxygenated monoterpenes, the oil suppressed germination and seedling development in *Bidens subalternans*, *Euphorbia heterophylla*, and *Macroptilium lathyroides* in a dose-dependent manner [50]. Germination of *B. subalternans* was completely suppressed at 0.5 and 1.0% concentrations, whereas *M. lathyroides* germination remained unaffected at these same rates. Post-emergence exposure caused visible foliar necrosis and chlorosis across species without altering total chlorophyll content, whereas cellular respiration was impaired exclusively in *E. heterophylla*.

### 3.19. Mentha *spp.*

*Mentha piperita* essential oil, characterized mainly by menthone and menthol, inhibited seed germination of *Solidago canadensis*, with the magnitude of inhibition varying with concentration [17]. In a comparative study, peppermint oil also suppressed germination of Mediterranean weeds, although its activity was weaker than that of cinnamon and lavender oils [35]. More pronounced herbicidal effects were observed against *Amaranthus retroflexus*, *Avena fatua*, *Portulaca oleracea*, and *Echinochloa crus-galli*, with activity increasing with concentration. At 20 µL mL^−1^, the oil completely inhibited *A. retroflexus* and *A. fatua*, whereas *P. oleracea* and *E. crus-galli* were less sensitive [51].

### 3.20. Ocimum *spp.*

*Ocimum basilicum* essential oil, rich in linalool (50.2%) and eugenol (15.5%), showed strong phytotoxic activity against *Portulaca oleracea*, *Chenopodium album*, *Solanum nigrum*, and *Echinochloa crus-galli*, suppressing seed germination, root development, and biomass accumulation [18]. More recent work with *O. gratissimum* essential oil, characterized predominantly by linalool, demonstrated inhibition of germination and seedling growth of *Lolium multiflorum*, including radicle and hypocotyl elongation and biomass accumulation, whereas the effects on the tested crop species were less pronounced [49]. In a cotton-swab assay using *Lactuca sativa* seedlings, *O. gratissimum* essential oil produced the strongest inhibition among the tested *Ocimum* oils, reducing radicle and hypocotyl elongation by 92.0% and 83.3%, respectively. *O. basilicum* and *O. citriodorum* oils also strongly inhibited radicle elongation, with inhibition of 77.6% [33].

### 3.21. Origanum *spp.*

*Origanum* essential oils have shown phytotoxic activity against both invasive and agricultural weeds. *O. vulgare* essential oil inhibited the germination of *Solidago canadensis*, with significant phytotoxicity observed at 1.25 µg mL^−1^ [17]. The oil also exhibited herbicidal activity against *Sinapis arvensis* and was dominated by carvacrol (34.0%) and *γ*-terpinene (21.6%), with *p*-cymene accounting for 9.4% of the composition [23]. In a recent *in planta* study, *O. majorana* essential oil nanoemulsion significantly suppressed germination and early growth of *Amaranthus retroflexus*, while producing comparatively limited effects on the tested crops [20].

### 3.22. Pelargonium *spp.*

The nanoemulsified *P. graveolens* essential oil exhibited dose-dependent inhibition of *A. retroflexus* germination and seedling development while maintaining relatively lower phytotoxicity toward *Z. mays* and *G. max* [20].

### 3.23. Petroselinum *spp.*

*Petroselinum crispum* essential oil showed moderate-to-low phytotoxicity in a comparative study of annual aromatic plants, reducing weed germination and early seedling development [18]. In contrast, parsley seed essential oil showed pronounced activity against *Lactuca sativa*, with 1,3,8-menthatriene (22.59%), apiole (22.41%), and *β*-phellandrene (15.02%) as the major constituents. At 0.5%, the oil strongly inhibited seed germination and root and hypocotyl growth, with effects comparable to those of 1% glyphosate [52]. Molecular docking further indicated stable interactions of *trans*-*p*-menth-6-en-2,8-diol with 5-enolpyruvylshikimate-3-phosphate synthase, although this computational result does not establish the mechanism of phytotoxicity.

### 3.24. Pimpinella anisum *L.* (Aniseed)

*Pimpinella anisum* essential oil, dominated by anethole (88.6%), showed strong inhibitory activity against *Solidago canadensis* seed germination, consistent with the pronounced phytotoxicity of anethole [17]. In a previous Petri-dish bioassay involving four weed species (*Portulaca oleracea*, *Chenopodium album*, *Solanum nigrum*, and *Echinochloa crus-galli*), *P. anisum* essential oil, rich in limonene (48.5%) and *trans*-anethole (40.4%), exhibited relatively low direct phytotoxicity, indicating limited inhibition of weed germination and seedling growth [18].

### 3.25. Perovskia abrotanoides *Karel.* (Perovskia)

*Perovskia abrotanoides* essential oil, characterized by camphor (22.8%), 1,8-cineole (18.7%), and *α*-pinene (13.7%) as the major constituents, showed pronounced phytotoxic activity in preliminary screening, inhibiting lettuce radicle growth by approximately 77.6% [33]. Its nanoemulsion also caused visible chlorosis, necrosis, and tip burn in the tested weeds and increased ion leakage. In *Bromus japonicus*, treatment reduced catalase activity by 52.32%, whereas SOD activity in *Festuca* spp. decreased by 41%.

### 3.26. Pinus *spp.*

Essential oils from *Pinus* species have shown phytotoxic activity against both weeds and crop species. The essential oil of the Balkan endemic *P. peuce* inhibited seed germination and early seedling development in preliminary phytotoxicity assays, indicating activity against the tested plant species [53]. *P. sylvestris* essential oil also inhibited seedling growth, although it showed the weakest overall phytotoxicity among the three commercial oils evaluated against weeds, crops, and invasive species [40]. In a field-relevant outdoor pot study, *P. sylvestris* oil reduced the dry weight of *Lolium rigidum* and *Avena sterilis* to 33% and 31–33% of the untreated controls, respectively, while producing a stronger effect against *Galium aparine*, for which dry weight and plant height were reduced to 14% and 18% of the control, respectively [36].

### 3.27. Piper *spp.*

Essential oils from *Piper* species have also demonstrated considerable phytotoxic and herbicidal potential. Essential oils of *Piper dilatatum* and *Piper divaricatum* showed strong herbicidal activity against *Lolium perenne*, while *P. aff. hispidum* reduced root growth of *Lactuca sativa*. Among the tested oils, *P. dilatatum* exhibited the strongest antifeedant activity and was characterized predominantly by apiol (79.0%), whereas *P. divaricatum* was rich in eugenol (37.5%) and methyl eugenol (36.3%) [55]. More recently, essential oils of *P. amalago* and *P. dilatatum* were formulated as nanoemulsions to overcome the poor water solubility and rapid degradation that limit the practical application of essential oils. The resulting nanoemulsions, characterized by nanometric droplet sizes (~10 nm), low polydispersity, and improved thermal stability, inhibited germination and seedling growth of the weeds *Bidens pilosa* and *Lolium multiflorum*, as well as the model species *Lactuca sativa*. The observed phytotoxicity was accompanied by cytogenotoxic and mutagenic effects and alterations in photosynthetic pigment contents, suggesting oxidative damage and disruption of chlorophyll metabolism [54]. These findings highlight both the intrinsic herbicidal potential of *Piper* essential oils and the potential of nanoformulation to improve their stability and practical applicability in weed management.

### 3.28. Salvia *spp.*

*Salvia officinalis* essential oil has demonstrated phytotoxic activity across a range of plant species, although its effects vary depending on the target species and developmental parameter assessed. The essential oil affected seed germination and seedling growth of *Triticum aestivum*, *Raphanus sativus*, *Solanum lycopersicum*, and *Trigonella foenum-graecum*, with differential effects on radicle and hypocotyl elongation, indicating species-dependent sensitivity [56]. Similarly, *S. officinalis* essential oil inhibited the germination of *Solidago canadensis*, although its activity was weaker than that of the most phytotoxic essential oils evaluated [17]. More recently, an essential oil rich in 1,8-cineole, *cis*-thujone, and *trans*-thujone inhibited both germination and seedling growth of *Sinapis arvensis*, *Trifolium campestre*, and *Lepidium sativum* at 4 µL mL^−1^, while also increasing malondialdehyde accumulation and electrolyte leakage, suggesting oxidative stress and disruption of membrane integrity [57].

### 3.29. Santolina chamaecyparissus *L.* (Cotton Lavender)

*Santolina chamaecyparissus* essential oil showed comparatively weaker and more selective herbicidal activity [51]. At 20 μL mL^−1^, it achieved approximately 90% control of *A. retroflexus*, whereas its effects against the other tested weeds were considerably lower. This suggests a narrower spectrum of activity than that observed for *T. capitata* and *M. piperita*.

### 3.30. Satureja *spp.*

Essential oils from wild and cultivated populations of *Satureja calamintha* were chemically characterized, and in both oils, pulegone (68.58–72.93%), menthone (10.15–12.07%), and menthol (6.31–9.83%) were the dominant compounds [58]. Both oils exhibited allelopathic activity by inhibiting weed germination and seedling growth, with particularly pronounced effects on *Carrichtera annua*. A nanoemulsion of *Satureja bachtiarica* essential oil, characterized by thymol (33.3%), carvacrol (25.7%), and *γ*-terpinene (16.4%), also strongly inhibited *Amaranthus retroflexus* germination and seedling growth, with greater effects at higher concentrations and comparatively limited effects on the tested crops [20].

### 3.31. Syzygium *spp.*

*Syzygium aromaticum* essential oil showed pronounced phytotoxic activity in both preliminary and subsequent assays. Clove bud essential oil applied at 5% (*v*/*v*) caused rapid injury and strongly suppressed weed growth [34]. In an initial lettuce assay, the oil inhibited hypocotyl growth by 55.7%, while its nanoemulsion produced 87.7% inhibition of lettuce radicle growth and caused visible foliar injury in the tested weeds [33]. The nanoemulsion induced yellowing, chlorosis, necrosis, and tip burn and resulted in more than 90% desiccation of *Bromus japonicus* within seven days.

### 3.32. Thymbra capitata *(L.) Cav.* (Spanish Oregano)

*Thymbra capitata* essential oil showed the strongest herbicidal activity among the three investigated oils, including *Mentha piperita* and *Santolina chamaecyparissus* [51]. At the highest tested concentration of 12 μL mL^−1^, it completely controlled *Avena fatua*, *Echinochloa crus-galli*, and *Amaranthus retroflexus*, while achieving approximately 90% control of *Portulaca oleracea*. Its herbicidal activity increased with increasing concentration.

### 3.33. Thymus *spp.*

Essential oils from Mediterranean *Thymus* species have shown strong phytotoxic activity against both weeds and crop species. Essential oils of *Thymus algeriensis*, *T. ciliatus* subsp. *coloratus*, and two chemotypes of *T. vulgaris* completely inhibited seed germination of *Lolium perenne* and *Amaranthus retroflexus* at 20 μL mL^−1^, while post-emergence treatments revealed differential sensitivity between the two weeds, with *A. retroflexus* being more susceptible than *L. perenne* [59]. Red thyme essential oil applied at 5% (*v*/*v*) also caused rapid and severe injury to several weed species, resulting in substantial growth suppression and biomass reduction [34]. The phytotoxicity of *T. vulgaris* essential oil was further demonstrated in wheat and barley, with complete inhibition of germination and seedling development at 90 μL. At 30 μL, germination and seedling development were completely suppressed in barley, while wheat germination was also completely inhibited. Even at 10 μL, the oil significantly reduced wheat germination and several seedling growth parameters. The oil was dominated by *p*-cymene (38.2%), thymol (25.6%), and *γ*-terpinene (13.6%) [31].

### 3.34. Trachyspermum ammi *L.* (Ajwain)

*Trachyspermum ammi* essential oil and its nanoemulsion have shown strong phytotoxic activity against weeds. The nanoemulsion reduced germination and growth of *Amaranthus retroflexus* while producing comparatively weaker effects on maize and soybean [20]. In a separate study, *T. ammi* essential oil, rich in thymol (61.3%), *γ*-terpinene (20.2%), and *o*-cymene (15.6%), strongly inhibited lettuce radicle growth in preliminary screening, whereas its nanoemulsion completely suppressed radicle development in the cotton-swab assay [33]. Against established weeds, application of the 1% nanoemulsion caused yellowing, chlorosis, necrosis, and tip burn, with more than 90% desiccation of *Bromus japonicus*, *Agropyron repens*, and *Festuca* spp. after seven days.

### 3.35. Zataria *spp.*

Nanoemulsions of *Zataria multiflora* and *Z. tenuior* essential oils strongly inhibited *Amaranthus retroflexus* germination and seedling growth [20]. The *Z. multiflora* nanoemulsion was characterized by carvacrol (50.8%) and thymol (30.0%) as the major constituents, whereas the *Z. tenuior* oil was dominated by thymol (33.8%) and pulegone (22.2%).

## 4. Mechanisms of Action

The phytotoxic effects of essential oils stem from multiple interconnected cellular targets rather than a single mode of action. Lipophilic constituents, primarily monoterpenes and phenylpropanoids, may affect membrane integrity by altering permeability and triggering electrolyte leakage. This disruption can be accompanied by an imbalance in reactive oxygen species (ROS), resulting in oxidative damage and impaired cellular function [13,32,66]. Experimental evidence also points to interference with photosynthetic machinery, hormonal regulation, reserve mobilization, and cell division. Specifically, essential oil constituents from *Artemisia fragrans* affected photosynthetic processes and cellular oxidative status [26], whereas monoterpenoids from *A. austriaca* altered the gibberellin and abscisic acid balance and inhibited enzymes involved in reserve mobilization during germination [24].

**Figure 1:**
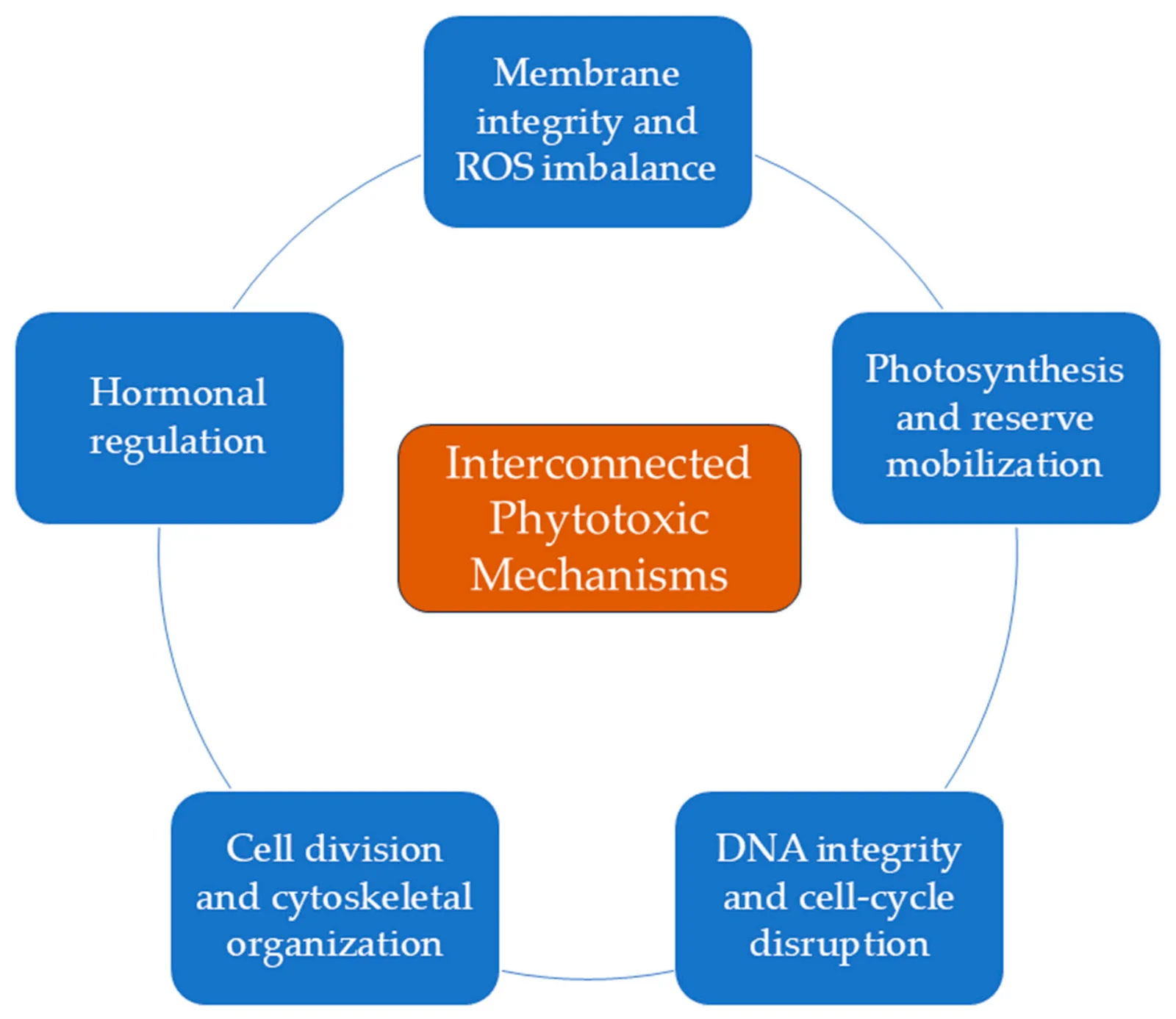
Proposed cellular and physiological mechanisms underlying essential oil phytotoxicity.

The main mechanisms associated with the phytotoxic effects of essential oils, including effects on membrane integrity, oxidative status, physiological and metabolic processes, and cellular and DNA integrity, are summarized in Figure 1.

At the cellular level, essential oils may also affect DNA integrity and cell-cycle progression. For instance, exposure to *Acorus calamus* essential oil was associated with oxidative stress, electrolyte leakage, and DNA damage, as well as disturbances in DNA replication during the S phase, while subsequent observations indicated alterations in mitosis and the cytoskeleton [62,63]. Thus, the observed inhibition of root and seedling growth may reflect the combined effects of disturbances occurring across several interconnected cellular pathways.

The severity and specific nature of these phytotoxic responses may also depend on exposure concentration. Increasing concentrations can progressively intensify physiological and cellular disturbances, although response thresholds vary among essential oils and target species. Concentration-response relationships are therefore influenced by the interplay of chemical composition, constituent interactions, developmental stage, and species susceptibility [11,13,34,66].

Evaluations of isolated compounds further clarify the contribution of individual constituents to essential oil phytotoxicity. Linalool, thymol, and carvacrol have each been reported to exert phytotoxic effects when tested individually. Linalool was identified as a major active constituent of *Artemisia absinthium* essential oil, with isolated linalool inhibiting root-hair formation and metaxylem development [21]. More recently, individual applications of linalool and eugenol in studies of *Ocimum* species inhibited lettuce germination and seedling growth [67]. Likewise, carvacrol and thymol inhibited seed germination and seedling growth in several weed species, including *Amaranthus retroflexus*, *Chenopodium album*, and *Rumex crispus* [68], while both compounds were also associated with alterations in the cell cycle of root meristematic cells [69]. These findings support the contribution of individual constituents to essential oil phytotoxicity, while also emphasizing that the activity of the complete essential oil cannot necessarily be attributed to its major component alone.

Among individual constituents, 1,8-cineole provides a well-documented example of direct phytotoxicity and allelopathic activity. When isolated from *Eucalyptus urophylla*, 1,8-cineole inhibited lettuce germination and seedling growth, with root elongation being particularly sensitive [70]. In *Brassica campestris*, it inhibited root growth, reduced the mitotic index in the root apical meristem, and suppressed DNA synthesis [71]. Further cellular studies in tobacco BY-2 cultures showed that 1,8-cineole inhibited both cell proliferation and elongation [72]. These findings illustrate how individual constituents may contribute to the cellular disturbances underlying the phytotoxic effects of essential oils.

## 5. Formulation and Delivery Strategies

**Figure 2:**
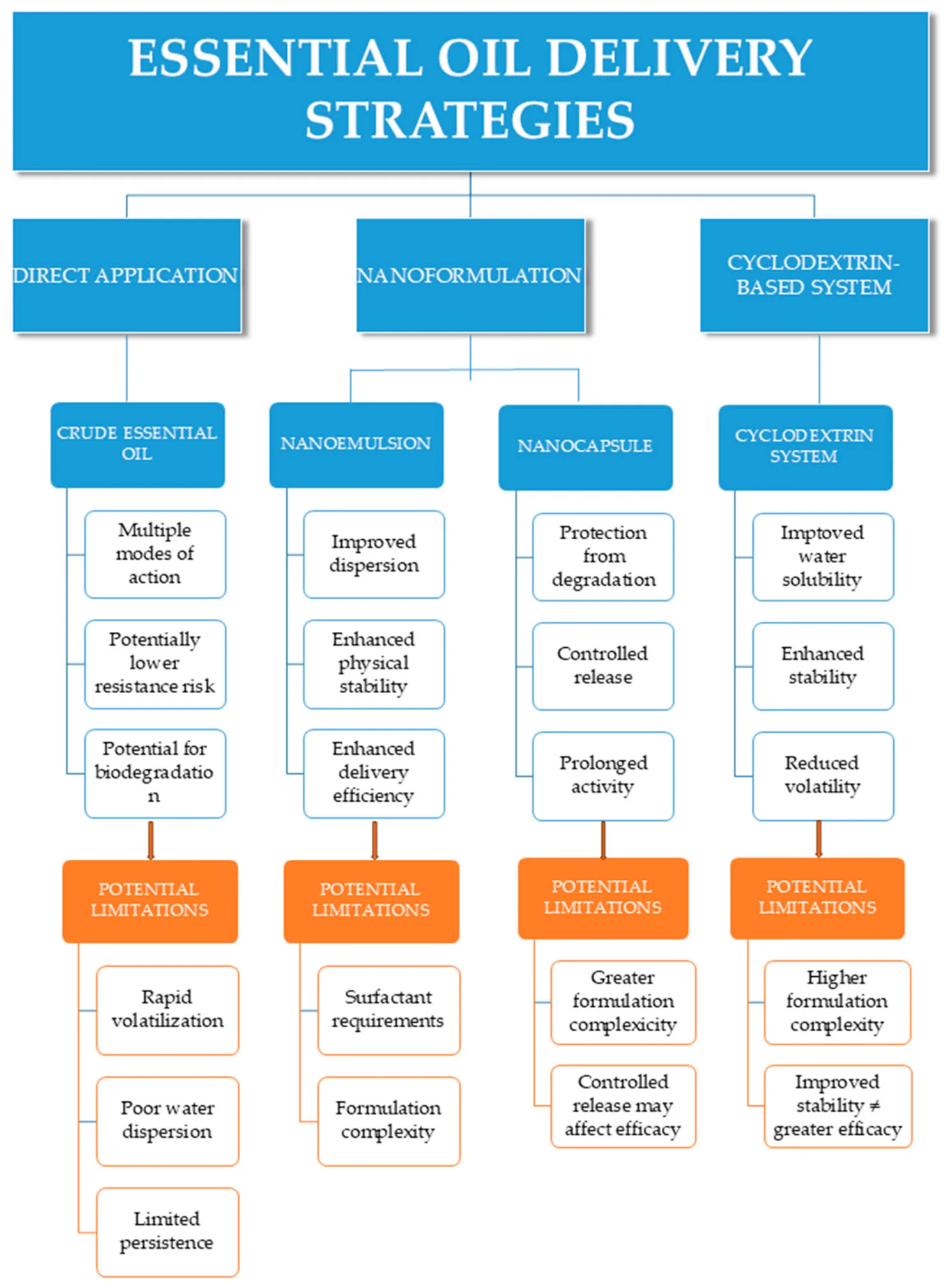
Comparative overview of essential oil delivery strategies for bioherbicide development.

The practical application of essential oils as bioherbicides is constrained by their physicochemical properties, including high volatility, poor water solubility, susceptibility to degradation, and limited persistence under field conditions [73,74]. These properties may reduce their dispersion, stability, retention on plant surfaces, and overall herbicidal efficacy. Consequently, formulation and delivery strategies have become important areas of research for improving the performance of essential oils under agronomically relevant conditions [73,75]. The main delivery approaches discussed in this section and their respective roles in improving the formulation and application of essential oils are summarized in Figure 2.

### 5.1. Limitations of Direct Essential Oil Application

Direct application of essential oils can result in rapid volatilization and degradation, while their hydrophobic nature limits uniform dispersion in aqueous spray systems [73,76]. These characteristics may reduce the stability, distribution, and persistence of essential oils following application, potentially limiting their herbicidal efficacy under field conditions [77]. In addition, the complex chemical composition of essential oils can influence their physicochemical properties and biological activity, making formulation and dose standardization particularly challenging [78].

### 5.2. Nanoemulsions and Encapsulation Systems

Nanoemulsions and related encapsulation systems have been investigated to improve the dispersion, stability, and delivery of essential oils. By reducing droplet size and modifying the physicochemical properties of the formulations, these systems may improve the handling and application of hydrophobic essential oils and, in some cases, enhance herbicidal efficacy [79]. Nanoemulsions primarily improve the dispersion and physical stability of hydrophobic essential oils, whereas nanocapsules can additionally provide a protective matrix and more controlled release of the encapsulated compounds [76,79]. However, formulation does not necessarily improve selectivity, and increased delivery efficiency may also increase exposure of crops and non-target organisms.

Several studies have reported enhanced phytotoxic activity following nanoformulation of essential oils from *Zataria multiflora*, *Pelargonium graveolens*, *Piper amalago*, *P. dilatatum*, *Trachyspermum ammi*, *Syzygium aromaticum*, *Cinnamomum cassia*, *Perovskia abrotanoides*, and *Cymbopogon* species [20,33,37,54]. Compared with the corresponding crude oils, these formulations showed improved dispersion and, in several cases, greater inhibition of weed germination or growth. However, the magnitude of the improvement varied according to essential oil composition, formulation characteristics, target species, and application conditions. For example, a *Cymbopogon nardus* nanoemulsion with a droplet size of approximately 79 nm showed good physical stability during storage and strong concentration-dependent inhibition of *Echinochloa crus-galli* germination and seedling growth [37]. Similarly, nanoemulsions containing *Piper amalago* and *P. dilatatum* essential oils exhibited nanoscale droplet sizes and greater thermal stability than the corresponding crude oils, while inhibiting the germination and early growth of *Bidens pilosa* and *Lolium multiflorum* [54]. These findings indicate that the benefits of nanoformulation depend not only on droplet size but also on essential oil composition, formulation properties, target species, and application conditions.

The potential of formulation strategies has also been demonstrated under conditions more closely related to practical weed management. Nanoemulsified thyme essential oil and *β*-cyclodextrin-based formulations provided greater suppression of *E. crus-galli* and *Portulaca oleracea* than the corresponding crude oil, while maize biomass remained higher than in the unweeded control at the tested concentrations [60]. Similarly, citronella essential oil nanoemulsions showed post-emergence activity against *E. crus-galli* and *Amaranthus tricolor*, accompanied by reductions in photosynthetic pigments and increased electrolyte leakage and malondialdehyde accumulation [37]. These findings indicate that formulation can improve the performance of essential oils beyond effects observed in seed germination assays, although efficacy remains strongly dependent on the target species, application rate, and formulation characteristics.

Importantly, enhanced biological activity was not always accompanied by improved safety. Cytogenotoxic and mutagenic effects were observed in *Lactuca sativa* following exposure to nanoemulsions containing *P. amalago* and *P. dilatatum* essential oils, demonstrating the need to evaluate formulation performance together with potential effects on crops and other non-target organisms [54]. Likewise, foliar symptoms including chlorosis, necrosis, and desiccation have been reported following application of several essential oil nanoemulsions [33,37]. Thus, nanoformulation should not be regarded as inherently improving selectivity or environmental safety. Rather, its main value lies in modifying the physicochemical and delivery properties of essential oils, while crop safety, non-target effects, and field performance must be assessed for each formulation.

### 5.3. Cyclodextrin-Based and Controlled Release Systems

Cyclodextrin-based formulations have been investigated as an alternative approach for improving the formulation properties of essential oils. An inclusion complex of *Psidium gaudichaudianum* leaf essential oil with 2-hydroxypropyl-*β*-cyclodextrin showed herbicidal activity against *Lolium multiflorum* and *Bidens pilosa*, while improving the thermal stability and water solubility of the essential oil [80]. However, complexation did not consistently increase herbicidal activity compared with the crude essential oil, indicating that improved formulation properties do not necessarily result in greater intrinsic phytotoxicity. Further evidence was obtained in pot experiments with maize, where *β*-cyclodextrin nanoencapsulated thyme essential oil was compared with the crude oil and a nanoemulsified formulation [60]. The formulated treatments provided greater suppression of *Echinochloa crus-galli* and *Portulaca oleracea* than the crude essential oil at the tested concentrations, while maize biomass was maintained or increased relative to the unweeded control. These results indicate that cyclodextrin-based formulation can improve the performance of some essential oils under pot conditions, although the response depends on the essential oil, formulation, target weed, and application conditions.

Overall, the available evidence indicates that cyclodextrin-based systems can modify the physicochemical properties, stability, and delivery of essential oils, although the effects on herbicidal efficacy may vary among formulations and essential oils [81,82]. Thus, improved stability or solubility does not necessarily translate into greater phytotoxicity, and direct comparison of crude and formulated oils under relevant application conditions is important for assessing their practical performance and crop safety [60,80].

The three formulation approaches offer different advantages. Nanoemulsions mainly improve dispersion and physical stability, whereas nanocapsules may provide greater protection against degradation and more controlled release. Cyclodextrin-based systems can improve the solubility and stability of essential oils and reduce their volatility. However, improved physicochemical properties do not necessarily result in greater herbicidal efficacy, and formulation performance depends on essential oil composition, formulation characteristics, target species, and application conditions. Compared with direct application of crude essential oils, advanced formulations may improve handling, stability, and delivery, but their practical adoption may also be limited by production costs, manufacturing complexity, scalability from laboratory to field applications, and environmental and regulatory considerations.

### 5.4. Crop Safety and Environmental Considerations

Improved stability and delivery may increase herbicidal efficacy, but they may also modify exposure patterns and persistence in agricultural environments [83]. Therefore, assessment of essential oil-based herbicides should consider crop tolerance, effects on non-target organisms, soil microbial communities, and environmental persistence in addition to weed suppression [84]. Such effects may depend strongly on essential oil type and application rate. For example, essential oils of *Thymbra capitata*, *Mentha piperita*, and *Santolina chamaecyparissus* showed concentration-dependent herbicidal activity against several weed species under pot conditions, while reported effects on soil microbial communities varied with essential oil type, concentration, and exposure duration [51]. Although most changes were transient under the conditions investigated, the highest concentration of *T. capitata* essential oil caused more persistent changes in microbial activity. These findings highlight the importance of establishing application rates that provide effective weed suppression while minimizing undesirable effects on crops and the soil environment [85]. Regulatory considerations are also important for the development of nanoformulated bioherbicides, as their assessment may need to address not only the active essential oil but also formulation components, particle characteristics, environmental fate, and potential exposure of non-target organisms [77,78]. Thus, practical evaluation of essential oil-based herbicides should extend beyond herbicidal efficacy to include crop selectivity, non-target effects, and environmental responses under relevant application conditions [86].

## 6. Current State, Research Gaps, and Future Perspectives

A major challenge for the development of essential oils as bioherbicides is the translation of laboratory phytotoxicity into reliable performance under agricultural conditions. Although individual constituents such as carvacrol, thymol, and eugenol can cause strong or complete inhibition of weed germination under laboratory conditions, their performance may be considerably lower under more realistic application conditions [11,12]. Laboratory assays therefore provide the most extensive evidence, whereas pot and particularly field studies remain comparatively limited, and strong laboratory activity does not necessarily translate into comparable weed control under agricultural conditions [12,34,36,40,47]. Future research should therefore move beyond laboratory germination assays towards a stepwise evaluation encompassing seedling, pot or greenhouse, and field experiments, while considering weed developmental stage, application method, essential oil composition, formulation, and application rate. Greater standardization of experimental protocols and dose reporting is also needed to enable meaningful comparison among studies and to establish reproducible dose–response relationships. Moreover, the herbicidal activity of complete essential oils cannot always be predicted from the activity of their major constituents considered individually, as interactions among constituents may contribute to the overall phytotoxic response. Chemical composition, concentration, target species, and developmental stage can therefore substantially influence the observed efficacy [11,12,34,66].

A better understanding of the mechanisms underlying essential oil phytotoxicity and weed selectivity represents another important research priority. Integrating physiological, biochemical, metabolomic, transcriptomic, and computational approaches could help clarify the cellular targets and pathways affected by essential oils and their major constituents and may support the identification of chemical profiles associated with more predictable herbicidal activity [13,20,24,32,62,63,87]. At the same time, formulation development should increasingly be integrated with crop safety, non-target effects, environmental persistence, and field efficacy rather than evaluated solely on the basis of improved delivery or herbicidal activity [83,84]. Crop selectivity represents another important consideration for the practical development of essential oil-based bioherbicides. Effective weed suppression should be evaluated together with potential phytotoxic effects on crops and other non-target plants, as the concentration required for weed control may overlap with that causing crop injury. Comparative studies involving target weeds and crops under the same experimental conditions are therefore needed to establish selectivity margins and identify suitable application strategies [40,46,48].

Environmental safety also requires greater attention before essential oil-based bioherbicides can be considered for large-scale application. Although essential oils are often regarded as environmentally favorable because of their natural origin and generally rapid degradation, their effects on non-target organisms, including beneficial soil microorganisms, insects, and other soil fauna, remain insufficiently characterized [51,84,85]. The persistence and degradation of individual essential oil constituents in soil may vary according to their chemical structure and environmental conditions, making their environmental fate difficult to predict. Large-scale and repeated applications may therefore produce effects that are not apparent in short-term laboratory assays, including alterations of soil microbial communities and unintended effects on non-target plants and organisms. Field studies under realistic application regimes are consequently needed to evaluate persistence, degradation, and potential ecological effects before wider agricultural adoption.

Finally, the practical adoption of essential oil-based bioherbicides will require economic feasibility, standardized formulations, appropriate regulatory frameworks, and integration with cultural, mechanical, biological, and precision-based weed management strategies. Valorization of aromatic plant residues and other underutilized biomass streams may further contribute to circular approaches, provided that recovery, formulation, and application are technically and economically feasible [8,88].

## 7. Conclusions

Essential oils show considerable potential as plant-derived bioherbicides, but current evidence indicates that strong phytotoxicity under laboratory conditions does not necessarily translate into reliable and selective weed control under field conditions, together with adequate environmental safety. Formulation and delivery strategies can improve their stability and performance but do not inherently overcome challenges related to crop safety, non-target effects, environmental persistence, and variability in efficacy. Therefore, the future development of essential-oil-based bioherbicides should focus not simply on demonstrating phytotoxic activity, but on achieving reproducible, field-validated, and agronomically relevant performance through standardized evaluation, appropriate formulation, crop selectivity, and environmental assessment. Essential oils should thus be viewed not as immediate replacements for conventional herbicides, but as promising components of integrated and sustainable weed management systems.

## Figures and Tables

**Table 1:** Examples of reported phytotoxic and herbicidal effects of essential oils on weed and cultivated species, including information on plant species, major constituents, experimental conditions, tested concentrations, target plants, and references.

Plant Species	Manin Compounds	Experiment	Tested Concentration	Target Plants	Reference
*Achillea millefolium*	camphor (11.5%), 1,8-cineole (8.9%), sabinene (7.9%)	*in vitro* (10 seeds/Petri dishes × 3)	0; 100; 250; 500; 1000 μg/mL	*Raphanus sativus* *Hordeum vulgare* *Lolium multiflorum* *Sinapis arvensis*	[14]
*Achillea millefolium*	germacrene D (1.1–46.6%), sabinene (4.0–38.9%), borneol (4.7–24.9%), camphor (0.6–17.6%), *α*-pinene (0.8–11.7%), artemisia ketone (3.1–12.9%)	*in vitro* (30 seeds/Petri dishes × 3)	0; 1; 2; 5 μL	*Lactuca sativa*	[15]
*Acorus calamus*	n.a.	*in vitro* (Petri dishes)	IC_50_: control, emulsifier, and essential oil	*Vicia faba* subsp. *minor* *Lupinus luteus* *Brassica napus* *Arabidopsis thaliana*	[16]
*Anethum graveolens*	limonene (35.8%), carvone (58.4%)	*in vitro* (100 seeds/Petri dishes × 3)	0; 2.5; 1.25; 0.625; 0.25; 0.125; 0.062 μg/mL	*Solidago canadensis*	[17]
*Anethum graveolens*	1,8-cineole (11.2%), *p*-cymene (7.1%)	*in vitro* (30 seeds/Petri dishes × 2)	0; 0.04; 0.08; 0.16; 0.32; 0.64 µL/mL	*Portulaca oleracea* *Chenopodium album* *Solanum nigrum* *Echinochloa crus-galli*	[18]
*Anethum graveolens*	*α*-phellandrene (35.7–51.4%), *p*-cymene (22.7–50.8%)	*in vitro* (10 seeds/Petri dishes × 3)	0; 0.5; 1.0; 1.5; 2.0 mg/mL	*Sinapis arvensis* *Trifolium campestre* *Lolium multiflorum*	[19]
*Anethum graveolens* nanoemulsions	carvone (59.2%), limonene (14.2%)	*in vitro* (20 seeds/Petri dishes × 4) *in vivo* (foliar application)	*in vitro*: 0; 50; 100; 300; 600; 900; 1200; 1500; 1800 µL/L *in vivo*: control and 2000 μL/L	*Amaranthus retroflexus* *Zea mays* *Glycine max*	[20]
*Artemisia absinthium*	1,8-cineole (25.6%), linalool (12.0%), *β*-myrcene (10.1%)	*in vitro* (10 seeds/Petri dishes × 3)	0; 0.25; 0.5; 1.0; 2.0; 5.0; 10.0 mg/mL	*Amaranthus retroflexus* *Medicago sativa* *Triticum aestivum* *Poa annua*	[21]
*Artemisia absinthium*	*trans*-thujone (18.9%), *cis*-ocimene epoxide (7.9%), bicyclogermacrene (7.0%)	*in vitro* (25 seeds/Petri dishes × 4)	0; 0.01; 0.025; 0.05; 0.10; 0.25; 0.50% *v*/*v*	*Amaranthus retroflexus* *Setaria viridis*	[22]
*Artemisia absinthium*	*trans*-thujone (35.6%), chamazulene (3.0%)	*in vitro* (30 seeds/Petri dishes × 3)	0; 0.5; 1.0; 1.5; 2.0 μL/mL	*Sinapis arvensis*	[23]
*Artemisia austriaca*	camphor (26.2%), 1,8-cineole (20.2%), *β*-myrcene (8.3%), borneol (6.3%)	*in vitro* (Petri dishes × 3)	0; 25; 50; 100; 150; 200 μg/mL	*Avena fatua*	[24]
*Artemisia campestris*	*β*-pinene (35.0%), 1,8-cineole (14.4%)	*in vitro* (plastic gearbox-type boxes)	0; 100; 1000; 2000 ppm	*Daucus carota* *Cicer arietinum* *Phaseolus vulgaris* *Triticum sativum*	[25]
*Artemisia fragrans*	*cis*-thujone (30.4%), camphor (26.4%), 1,8-cineole (12.6%), *trans*-thujone (10.0%)	*in vitro* (Petri dishes × 3) *in vivo* (pot experiments)	*in vitro*: 0; 1; 2; 3; 4; 5 μL/mL *in vivo*: 0; 0.5; 1.0; 2.0; 4.0% administered at rate of 100 mL/pot	*Convolvulus arvensis*	[26]
*Artemisia herba-alba*	camphor (26.0%), *cis*-thujone (9.6%), *trans*-thujone (8.4%), 1,8-cineole (8.0%), piperitenone (5.4%),	*in vitro* (10 seeds/Petri dishes × 3)	0; 125; 250; 500; 1000 μg/mL	*Lolium multiflorum* *Trifolium pratense* *Brassica napus* *Hordeum vulgare*	[27]
*Artemisia lancea*	1,8-cineole (18.7%)	*in vitro* (10 seeds/Petri dishes × 5)	0; 0.25; 0.5; 1.0; 2.0; 5.0 mg/mL	*Amaranthus retroflexus* *Setaria viridis*	[28]
*Artemisia scoparia*	*p*-cymene (20.5%), myrcene (14.0%), limonene (12.5%)	*in vitro* (25 seeds/Petri dishes × 5) *in vivo* (pot experiment)	*in vitro*: 0; 5; 10; 25; 50 µg EO/g *in vivo*: 0; 2; 4; 6% (*v*/*v*) foliar application	*Achyranthes aspera* *Cassia occidentalis* *Parthenium hysterophorus* *Echinochloa crus-galli* *Ageratum conyzoides*	[29]
*Artemisia vulgaris*	*cis*-sabinol (15.2%), *trans*-sabinyl acetate (11.1%), 1,8-cineole (10.9%), *trans*-piperitol (8.4%)	*in vitro* (20 seeds/Petri dishes × 3)	0; 0.25, 0.5, 1, 2 and 5 mg/mL	*Setaria viridis* *Poa annua* *Medicago sativa* *Amaranthus retroflexus*	[30]
*Artemisia vulgaris*	gurjunene (10.4%), *cis*-chrysanthenyl acetate (7.2%), *γ*-humulene (6.7%)	*in vitro* (25 seeds/Petri dishes × 4)	0; 0.01; 0.025; 0.05; 0.10; 0.25; 0.50% *v*/*v*	*Amaranthus retroflexus* *Setaria viridis*	[22]
*Chrysanthemum balsamita*	camphor (43.7%), *trans*-thujone (32.4%), camphene (12.0%)	*in vitro* (Petri dishes × 4)	0; 10; 30; 90 μL	*Hordeum vulgare* *Triticum aestivum*	[31]
*Cinnamomum cassia*	*trans*-cinnamaldehyde (75.0%)	*in vivo* (pot experiment)	0; 1; 3; 6%	*Trifolium incarnatum* *Lolium perenne*	[32]
*Cinnamomum cassia* nanoemulsion	cinnamaldehyde (86.9%)	*in vitro* (Dish pack method, Cotton swab method)	0; 1%	*Agropyron repens* *Bromus japonicus* *Chenopodium album* *Festuca* spp. *Lactuca sativa*	[33]
*Cinnamomum* sp.	n.a.	*in vivo* (pot experiment)	0; 5% (*v*/*v*)	*Artemisia vulgaris* *Glebionis coronaria* *Sylibum marianum* *Verbascum densiflorum* *Eleusine indica* *Ipomoea hederacea* *Jacquemontia tamnifolia* *Digitaria sanguinalis* *Sesbania exaltata*	[34]
*Cinnamomum zeylanicum*	n.a.	*in vitro* (25 seeds/Petri dishes × 4) *in vivo* (pot experiment)	*in vitro*: 0; 0.2; 0.6; 1.8; 5.4 mg/L *in vivo*: 0; 5.4; 21.6; 86.4, 345.6 mg/L	*Amaranthus retroflexus* *Solanum nigrum* *Portulaca oleracea* *Chenopodium album* *Sinapis arvensis* *Lolium* spp. *Vicia sativa*	[35]
*Coriandrum sativum*	1,8-cineole (36.9%), linalool (28.7%), limonene (20.2%)	*in vitro* (30 seeds/Petri dishes × 2)	0; 0.04; 0.08; 0.16; 0.32; 0.64 µL/mL	*Portulaca oleracea* *Chenopodium album* *Solanum nigrum* *Echinochloa crus-galli*	[18]
*Cuminum cyminum*	cumin aldehyde (49.6%), *α*-terpinen-7-al (11.6%), *p*-cymene (10.4%)	*in vitro* (Petri dishes × 4)	0; 10; 30; 90 μL	*Hordeum vulgare* *Triticum aestivum*	[31]
*Cymbopogon citratus*	n.a.	*in vitro* (Petri dishes × 4) *in vivo* (pot experiment)	0; 5% (1000 mL/ha)	*Lolium rigidum* *Avena sterilis* *Galium aparine*	[36]
*Cymbopogon citratus*	neral (29.2%), geranial (18.2%)	*in vitro* (30 seeds/Petri dishes × 3)	0; 0.5; 1.0; 1.5; 2.0 μL/mL	*Sinapis arvensis*	[23]
*Cymbopogon nardus* nanoemulsion	citronellol (35.2%), geraniol (21.9%), citronellal (13.6%)	*in vivo* (pot experiment)	0; 2.5; 5; 10; 20; 40 mL/L	*Echinochloa crus-galli* *Amaranthus tricolor*	[37]
*Cymbopogon nardus*	citronellal (36.5%), geraniol (25.6%)	*in vivo* (plastic boxes)	0; 1; 10; 20%	*Digitaria horizontalis* *Cenchrus echinatus*	[38]
*Echinophora cinerea*	n.a.	*in vitro* (50 seeds/Petri dishes × 3)	0; 0.5; 1; 2; 5 μL/mL	*Chenopodium album*	[39]
*Eucalyptus citriodora*	citronellal (88.0%)	*in vitro* (20 seeds/Petri dishes × 5)	0; 0.125; 0.25; 0.50; 1 µL/mL	*Portulaca oleracea* *Lolium multiflorum* *Echinochloa crus-galli* *Solanum lycopersicum* *Cucumus sativus* *Nicotiana glauca*	[40]
*Eucalyptus citriodora*	citronellal (61.8%), isopulegol (11.9%)	*in vivo* (plastic boxes)	0; 1; 10; 20%	*Digitaria horizontalis* *Cenchrus echinatus*	[38]
*Eucalyptus cladocalyx*	spathulenol (21.6%), 1,8-cineole (20.5%), *p*-cymene (15.1%)	*in vitro* (30 seeds/Petri dishes × 3)	0; 0.5; 1.0; 1.5; 2.0 μL/mL	*Sinapis arvensis*	[23]
*Eucalyptus* sp.	1,8-cineole (32.4–70.8%)	*in vitro* (10 seeds/Petri dishes × 3)	0; 100; 250; 500; 1000 μg/mL	*Lolium multiflorum* *Sinapis arvensis* *Raphanus sativus* *Cucumis sativus*	[41]
*Foeniculum vulgare*	estragole/anisole (40.1%), anethole (38.1%), fenchone (16.5%)	*in vitro* (100 seeds/Petri dishes × 3)	0; 2.5; 1.25; 0.625; 0.25; 0.125; 0.062 μg/mL	*Solidago canadensis*	[17]
*Foeniculum vulgare*	*trans*-anethole (58.4%), limonene (16.0%)	*in vitro* (30 seeds/Petri dishes × 2)	0; 0.04; 0.08; 0.16; 0.32; 0.64 µL/mL	*Portulaca oleracea* *Chenopodium album* *Solanum nigrum* *Echinochloa crus-galli*	[18]
*Hesperozygis ringens*	pulegone (70.7%), limonene (4.5%)	*in vitro* (50 seeds/Petri dishes × 4) *in vivo* (vases experiment)	0; 1; 3; 6%	*Amaranthus viridis*	[42]
*Hesperozygis ringens*	pulegone	*in vitro* (20 seeds/Petri dishes × 5) *in vivo* (pot experiment)	*in vitro*: 0; 0.001; 0.0025; 0.005; 0.01; 0.02% *in vivo*: 0; 1.0%	*Bidens pilosa* *Paspalum notatum*	[43]
*Hesperozygis ringens*	pulegone (70.7%)	*in vitro* (20 seeds/Petri dishes × 5) *in vivo* (pot experiment, field experiment)	*Petri dishes*: 0; 0.001; 0.0025; 0.005; 0.01; 0.02% *Adult plants*: 0; 0.02; 0.04% *Field experiment:* 0; 1.0%	*Lolium multiflorum*	[44]
*Hesperozygis ringens*	pulegone (81.2%)	*in vitro* (20 seeds/Petri dishes × 4) *in vivo* (pot experiment)	*in vitro*: 0; 0.001; 0.0025; 0.005; 0.01%, *in vivo*: 0; 0.02% (*v*/*v*)	*Eragrostis plana*	[45]
*Hesperozygis ringens*	pulegone	*in vitro* (20 seeds/Petri dishes × 4) *in vivo* (pot experiment)	*in vitro*: 0; 0.001; 0.0025; 0.005; 0.01; 0.02%, *in vivo*: 0; 1.0%	*Conyza bonariensis* *Plantago australis*	[46]
*Hyssopus officinalis*	*cis*-pinocamphone (39.8%), *trans*-pinocamphone (9.8%), *β*-pinene (11.4%)	*in vitro* (Petri dishes × 4)	0; 10; 30; 90 μL	*Hordeum vulgare* *Triticum aestivum*	[31]
*Lavandula angustifolia*	linalool (38.7%), 1,8-cineole (26.5%), camphor (14.2%)	*in vitro* (20 seeds/Petri dishes × 5)	0; 0.125; 0.25; 0.50; 1 µL/mL	*Portulaca oleracea* *Lolium multiflorum* *Echinochloa crus-galli* *Solanum lycopersicum* *Cucumus sativus* *Nicotiana glauca*	[40]
*Lavandula angustifolia*	linalool (34.84%), menthyl acetate (28.10%)	*in vivo* (pot experiment)	0; 0.05; 0.10; 0.20; 0.30 g/mL	*Echinochloa crus-galli* *Galium aparine*	[47]
*Lavandula angustifolia*	linalyl acetate (41.0%), linalool (36.81%)	*in vivo* (pot experiment)	0; 0.5; 1.0; 2.0%	*Lactuca sativa*	[48]
*Lavandula angustifolia*	linalool acetate (47.0%), linalool (28.4%)	*in vitro* (Petri dishes × 4)	0; 10; 30; 90 μL	*Hordeum vulgare* *Triticum aestivum*	[31]
*Lavandula* sp.	n.a.	*in vitro* (25 seeds/Petri dishes × 4) *in vivo* (pot experiment)	*in vitro*: 0; 0.2; 0.6; 1.8; 5.4 mg/L *in vivo*: 0; 5.4; 21.6; 86.4, 345.6 mg/L	*Amaranthus retroflexus* *Solanum nigrum* *Portulaca oleracea* *Chenopodium album* *Sinapis arvensis* *Lolium* spp. *Vicia sativa*	[35]
*Leptospermum scoparium*	n.a.	*in vitro* (Petri dishes × 4) *in vivo* (pot experiment)	0; 5% (1000 mL/ha)	*Lolium rigidum* *Avena sterilis* *Galium aparine*	[36]
*Levisticum officinale*	*α*-terpinyl acetate (64.8%), *β*-phellandrene (14.7%)	*in vitro* (Petri dishes × 4)	0; 10; 30; 90 μL	*Hordeum vulgare* *Triticum aestivum*	[31]
*Lippia alba*	eugenol (53.3%), *trans*-*β*-ocimene (13.4%),	*in vitro* (10 seeds/Petri dishes × 3)	0; 100; 200; 400; 800 µg/mL	*Hordeum vulgare* *Raphanus sativus* *Lolium multiflorum* *Sinapis arvensis*	[49]
*Lippia origanoides*	camphor (34.0%), camphene (11.0%), *β*-bisabolene (10.8%)	*in vitro* (25 seeds/Petri dishes × 4) *in vivo* (pot experiment)	0; 0.01; 0.05; 0.1; 0.5; 1.0%	*Bidens subalternans* *Euphorbia heterophylla* *Macroptilium lathyroides*	[50]
*Lippia sidoides*	thymol (61.7%), caryophyllene (10.7%)	*in vitro* (10 seeds/Petri dishes × 3)	0; 12.5; 25.0; 50.0 µg/mL	*Hordeum vulgare* *Raphanus sativus* *Lolium multiflorum* *Sinapis arvensis*	[49]
*Mentha piperita*	n.a.	*in vitro* (25 seeds/Petri dishes × 4) *in vivo* (pot experiment)	*in vitro*: 0; 0.2; 0.6; 1.8; 5.4 mg/L *in vivo*: 0; 5.4; 21.6; 86.4, 345.6 mg/L	*Amaranthus retroflexus* *Solanum nigrum* *Portulaca oleracea* *Chenopodium album* *Sinapis arvensis* *Lolium* spp. *Vicia sativa*	[35]
*Mentha piperita*	menthone (22.4%), menthol (49.3%)	*in vitro* (100 seeds/Petri dishes × 3)	0; 2.5; 1.25; 0.625; 0.25; 0.125; 0.062 μg/mL	*Solidago canadensis*	[17]
*Mentha piperita*	menthol (51.81%)	*in vivo* (pot experiment)	0; 12; 16; 20 μL/mL	*Avena fatua* *Echinochloa crus-galli* *Amaranthus retroflexus* *Portulaca oleracea*	[51]
*Ocimum basilcum*	linalool (50.2%), eugenol (15.5%)	*in vitro* (30 seeds/Petri dishes × 2)	0; 0.04; 0.08; 0.16; 0.32; 0.64 µL/mL	*Portulaca oleracea* *Chenopodium album* *Solanum nigrum* *Echinochloa crus-galli*	[18]
*Ocimum gratissimum*	linalool (62.7%), 1,8-cineole (7.1%)	*in vitro* (10 seeds/Petri dishes × 3)	0; 50; 100; 200; 400 µg/mL	*Hordeum vulgare* *Raphanus sativus* *Lolium multiflorum* *Sinapis arvensis*	[49]
*Origanum majorana*	*γ*-terpinene (13.5%), *cis*-sabinene hydrate (15.8%), terpinen-4-ol (27.0%)	*in vitro* (20 seeds/Petri dishes × 4) *in vivo* (foliar application)	*in vitro*: 0; 50; 100; 300; 600; 900; 1200; 1500; 1800 µL/L *in vivo*: control and 2000 μL/L	*Amaranthus retroflexus* *Zea mays* *Glycine max*	[20]
*Origanum vulgare*	carvacrol (34.0%), *γ*-terpinene (21.6%)	*in vitro* (30 seeds/Petri dishes × 3)	0; 0.5; 1.0; 1.5; 2.0 μL/mL	*Sinapis arvensis*	[23]
*Origanum vulgare*	carvacrol (78.2%)	*in vitro* (100 seeds/Petri dishes × 3)	0; 2.5; 1.25; 0.625; 0.25; 0.125; 0.062 μg/mL	*Solidago canadensis*	[17]
*Pelargonium graveolens*	citronellol (32.1%), geraniol (14.6%)	*in vitro* (20 seeds/Petri dishes × 4) *in vivo* (foliar application)	*in vitro*: 0; 50; 100; 300; 600; 900; 1200; 1500; 1800 µL/L *in vivo*: control and 2000 μL/L	*Amaranthus retroflexus* *Zea mays* *Glycine max*	[20]
*Perovskia abrotanoides* nanoemulsion	camphor (22.8%), 1,8-cineole (18.7%), *α*-pinene (13.7%)	*in vitro* (Dish pack method, Cotton swab method)	0; 1%	*Agropyron repens* *Bromus japonicus* *Chenopodium album* *Festuca* spp. *Lactuca sativa*	[33]
*Petroselinum crispum*	eugenol (14.1%), 1,8-cineole (10.8%)	*in vitro* (30 seeds/Petri dishes × 2)	0; 0.04; 0.08; 0.16; 0.32; 0.64 µL/mL	*Portulaca oleracea* *Chenopodium album* *Solanum nigrum* *Echinochloa crus-galli*	[18]
*Petroselinum crispum*	1,3,8-*p*-menthatriene (22.6%), apiole (22.4%), *β*-phellandrene (15.0%)	*in vitro* (20 seeds/Petri dishes × 5)	0; 0.1; 0.5; 1.0%	*Lactuca sativa*	[52]
*Pimpinella anisum*	anethole (88.6%)	*in vitro* (100 seeds/Petri dishes × 3)	0; 2.5; 1.25; 0.625; 0.25; 0.125; 0.062 μg/mL	*Solidago canadensis*	[17]
*Pimpinella anisum*	limonene (48.5%), *trans*-anethole (40.4%)	*in vitro* (30 seeds/Petri dishes × 2)	0; 0.04; 0.08; 0.16; 0.32; 0.64 µL/mL	*Portulaca oleracea* *Chenopodium album* *Solanum nigrum* *Echinochloa crus-galli*	[18]
*Pinus peuce*	*α*-pinene (10.5–56.0%), *β*-pinene (4.2–18.3%), limonene (1.2–60.0%)	*in vitro* (20 seeds/Petri dishes × 3)	0; 5; 10; 20 μL	*Papaver rhoeas* *Anthemis arvensis*	[53]
*Pinus sylvestris*	n.a.	*in vitro* (Petri dishes × 4) *in vivo* (pot experiment)	0; 5% (1000 mL/ha)	*Lolium rigidum* *Avena sterilis* *Galium aparine*	[36]
*Pinus sylvestris*	*α*-pinene (25.6%), *β*-pinene (15.9%), camphene (6.4%)	*in vitro* (20 seeds/Petri dishes × 5)	0; 0.125; 0.25; 0.50; 1 µL/mL	*Portulaca oleracea* *Lolium multiflorum* *Echinochloa crus-galli* *Solanum lycopersicum* *Cucumus sativus* *Nicotiana glauca*	[40]
*Piper* sp. nanoemulsions	*β*-elemene (5.2–17.8%) germacrene A (0–17.6%), linalool (0–12.1%), viridiflorene (0–22.9%) germacrene D (0–12.8%)	*in vitro* (25 seeds/Petri dishes × 5) *in vivo* (tubes in greenhouse)	0; 187.5; 375; 750; 1500; 3000 μg/mL	*Bidens pilosa* *Lolium multiflorum* *Lactuca sativa*	[54]
*Piper* sp.	*δ*-3 carene (0–35.3%) *p*-cymene (0–10.9%) limonene (0–27.1%) 1,8-cineole (0–37.5%) methyleugenol (0–36.3%) *γ*-elemene (0–10.7%) apiol (0–79.0%)	*in vitro* (100 seeds/12-well microplates)	dilution up to final concentrations 0.2 mg/mL	*Lactuca sativa* *lolium perenne*	[55]
*Salvia officinalis*	*trans*-thujone (34.2%), camphor (19.8%), limonene (15.0%)	*in vitro* (100 seeds/Petri dishes × 3)	0; 2.5; 1.25; 0.625; 0.25; 0.125; 0.062 μg/mL	*Solidago canadensis*	[17]
*Salvia officinalis*	camphor (25.14%), *cis*-thujone (18.83%), 1,8-cineole (14.14%)	*in vitro* (Petri dishes)	n.a.	*Triticum aestivum* *Raphanus sativus* *Solanum lycopersicum* *Trigonella foenum-graecum*	[56]
*Salvia officinalis*	1,8-cineole (26.4–17.5%), *cis*-thujone (11.9–9.56%), *trans*-thujone (22.9–10.33%)	*in vitro* (Petri dishes)	0; 1; 2; 3; 4 μL/mL	*Sinapis arvensis* *Trifolium campestre* *Lepidium sativum*	[57]
*Santolina chamaecyparissus*	1,8-cineole (17.5%)	*in vivo* (pot experiment)	0; 12; 16; 20 μL/mL	*Avena fatua* *Echinochloa crus-galli* *Amaranthus retroflexus* *Portulaca oleracea*	[51]
*Satureja bachtiarica*	thymol (33.3%), carvacrol (25.7%), *γ*-terpinene (16.4%)	*in vitro* (20 seeds/Petri dishes × 4) *in vivo* (foliar application)	*in vitro*: 0; 50; 100; 300; 600; 900; 1200; 1500; 1800 µL/L *in vivo*: control and 2000 μL/L	*Amaranthus retroflexus* *Zea mays* *Glycine max*	[20]
*Satureja calamintha*	pulegone (72.93–68.58%), menthone (12.07–10.15%), menthol (6.31–9.83%)	*in vitro* (30 seeds/Petri dishes × 5)	0; 0.12; 0.25; 0.50; 1; 2 µL/mL	*Notoceras bicorne* *Carrichtera annua*	[58]
*Syzygium aromaticum*	n.a.	*in vivo* (pot experiment)	0; 5% (*v*/*v*)	*Artemisia vulgaris* *Glebionis coronaria* *Sylibum marianum* *Verbascum densiflorum* *Eleusine indica* *Ipomoea hederacea* *Jacquemontia tamnifolia* *Digitaria sanguinalis* *Sesbania exaltata*	[34]
*Syzygium aromaticum* nanoemulsion	n.a.	*in vitro* (Dish pack method, Cotton swab method)	0; 1%	*Agropyron repens* *Bromus japonicus* *Chenopodium album* *Festuca* spp. *Lactuca sativa*	[33]
*Thymbra capitata*	carvacrol (72.3%)	*in vivo* (pot experiment)	0; 4; 8; 12 μL/mL	*Avena fatua* *Echinochloa crus-galli* *Amaranthus retroflexus* *Portulaca oleracea*	[51]
*Thymus* sp.	n.a.	*in vivo* (pot experiment)	0; 5% (*v*/*v*)	*Artemisia vulgaris* *Glebionis coronaria* *Sylibum marianum* *Verbascum densiflorum* *Eleusine indica* *Ipomoea hederacea* *Jacquemontia tamnifolia* *Digitaria sanguinalis* *Sesbania exaltata*	[34]
*Thymus* sp.	*α*-pinene (0.2–19.7%), linalool (2.5–93.1%), borneol (0–11.3%), *p*-cimene (0.1–35.6%), thymol (0–21.8%), carvacrol (0–18.2%)	*in vivo* (nursery trays under greenhouse conditions)	0; 5; 7.5; 10; 20 µL/mL	*Lolium perenne* *Amaranthus retroflexus*	[59]
*Thymus* sp. nanoemulsion	thymol (31.79), *γ*-terpinene (14.76%), *p*-cymene (12.09%)	*in vitro* (pot experiments)	0; 3; 6; 9% *v*/*v*	*Zea mays* *Echinochloa crus-galli* *Portulaca oleracea*	[60]
*Thymus vulgaris*	*p*-cymene (38.1%), thymol (25.6%), *γ*-terpinene (13.6%)	*in vitro* (Petri dishes × 4)	0; 10; 30; 90 μL	*Hordeum vulgare* *Triticum aestivum*	[31]
*Trachyspermum ammi*	thymol (35.8%), *γ*-terpinene (34.2%), *p*-cymene (22.0%)	*in vitro* (20 seeds/Petri dishes × 4) *in vivo* (foliar application)	*in vitro*: 0; 50; 100; 300; 600; 900; 1200; 1500; 1800 µL/L *in vivo*: control and 2000 μL/L	*Amaranthus retroflexus* *Zea mays* *Glycine max*	[20]
*Trachyspermum ammi* nanoemulsion	thymol (61.3%), *γ*-terpinene (20.2%), *o*-cymene (15.6%)	*in vitro* (Dish pack method, Cotton swab method)	0; 1%	*Agropyron repens* *Bromus japonicus* *Chenopodium album* *Festuca* spp. *Lactuca sativa*	[33]
*Zataria multiflora*	carvacrol (50.8%), thymol (30.0%)	*in vitro* (20 seeds/Petri dishes × 4) *in vivo* (foliar application)	*in vitro*: 0; 50; 100; 300; 600; 900; 1200; 1500; 1800 µL/L *in vivo*: control and 2000 μL/L	*Amaranthus retroflexus* *Zea mays* *Glycine max*	[20]
*Ziziphora tenuior*	thymol (33.8%), pulegone (22.2%)	*in vitro* (20 seeds/Petri dishes × 4) *in vivo* (foliar application)	*in vitro*: 0; 50; 100; 300; 600; 900; 1200; 1500; 1800 µL/L *in vivo*: control and 2000 μL/L	*Amaranthus retroflexus* *Zea mays* *Glycine max*	[20]

n.a.—not ariciable.

## Data Availability

No new data were created or analyzed in this study. Data sharing is not applicable to this article.
